# Synthesis, Anticonvulsant, and Antinociceptive Activity of New 3-(2-Chlorophenyl)- and 3-(3-Chlorophenyl)-2,5-dioxo-pyrrolidin-1-yl-acetamides

**DOI:** 10.3390/molecules26061564

**Published:** 2021-03-12

**Authors:** Małgorzata Góra, Anna Czopek, Anna Rapacz, Anna Gębska, Katarzyna Wójcik-Pszczoła, Elżbieta Pękala, Krzysztof Kamiński

**Affiliations:** 1 Department of Medicinal Chemistry, Faculty of Pharmacy, Jagiellonian University Medical College, Medyczna 9 St., 30-688 Krakow, Poland; malgorzata.gora@doctoral.uj.edu.pl (M.G.); k.kaminski@uj.edu.pl (K.K.); 2 Department of Pharmacodynamics, Faculty of Pharmacy, Jagiellonian University Medical College, Medyczna 9 St., 30-688 Krakow, Poland; anna2.gebska@student.uj.edu.pl; 3 Department of Pharmaceutical Biochemistry, Faculty of Pharmacy, Jagiellonian University Medical College, Medyczna 9 St., 30-688 Krakow, Poland; katarzynaanna.wojcik@uj.edu.pl (K.W.-P.); elzbieta.pekala@uj.edu.pl (E.P.)

**Keywords:** anticonvulsant activity, antinociceptive activity, pyrrolidine-2,5-dione, amides

## Abstract

The new series of 3-(2-chlorophenyl)- and 3-(3-chlorophenyl)-pyrrolidine-2,5-dione-acetamide derivatives as potential anticonvulsant and analgesic agents was synthesized. The compounds obtained were evaluated in the following acute models of epilepsy: maximal electroshock (MES), psychomotor (6 Hz, 32 mA), and subcutaneous pentylenetetrazole (*sc*PTZ) seizure tests. The most active substance-3-(2-chlorophenyl)-1-{2-[4-(4-fluorophenyl)piperazin-1-yl]-2-oxoethyl}-pyrrolidine-2,5-dione (**6**) showed more beneficial ED_50_ and protective index values than the reference drug—valproic acid (68.30 mg/kg vs. 252.74 mg/kg in the MES test and 28.20 mg/kg vs. 130.64 mg/kg in the 6 Hz (32 mA) test, respectively). Since anticonvulsant drugs are often effective in neuropathic pain management, the antinociceptive activity for two the promising compounds—namely, **6** and **19**—was also investigated in the formalin model of tonic pain. Additionally, for the aforementioned compounds, the affinity for the voltage-gated sodium and calcium channels, as well as GABA_A_ and TRPV1 receptors, was determined. As a result, the most probable molecular mechanism of action for the most active compound **6** relies on interaction with neuronal voltage-sensitive sodium (site 2) and L-type calcium channels. Compounds **6** and **19** were also tested for their neurotoxic and hepatotoxic properties and showed no significant cytotoxic effect.

## 1. Introduction

Epilepsy is a chronic neurological disorder that affects approximately 50 million people worldwide. According to the International League Against Epilepsy, the current classification of seizure types distinguishes three major groups as follows: generalized onset seizures (motor or absence), focal onset seizures (which may include abnormal behaviors, responsiveness, sensations, or movements), and unknown onset seizures [1,2]. A seizure is defined as an interruption in neurologic function caused by abnormal neuronal signaling in the brain. Despite numerous antiepileptic drugs (AEDs) available, it is still widely recognized that nearly a third of all people with epilepsy do not achieve satisfying seizures control with existing medications [3,4]. Moreover, in case of *status epilepticus*, which leads to abnormally prolonged seizures, the consequences depending on the type and duration of seizures may be fatal, including neuronal death, neuronal injury, and alteration of neuronal networks. Thus, there is a significant unmet clinical need to find adequate and effective treatment of especially pharmacoresistant epilepsy.

Apart from epilepsy, AEDs (i.e., pregabalin, gabapentin, carbamazepine) are also extensively used in the treatment of central and peripheral neuropathic pain (including cancer-related neuropathy, painful diabetic neuropathy, postherpetic neuralgia, trigeminal neuralgia, and also in chronic pain conditions such as fibromyalgia or migraine prophylaxis), for which management is difficult when applying the conventional analgesics such as paracetamol, nonsteroidal anti-inflammatory drugs (NSAIDs), or opioids [5]. 

**Figure 1:**
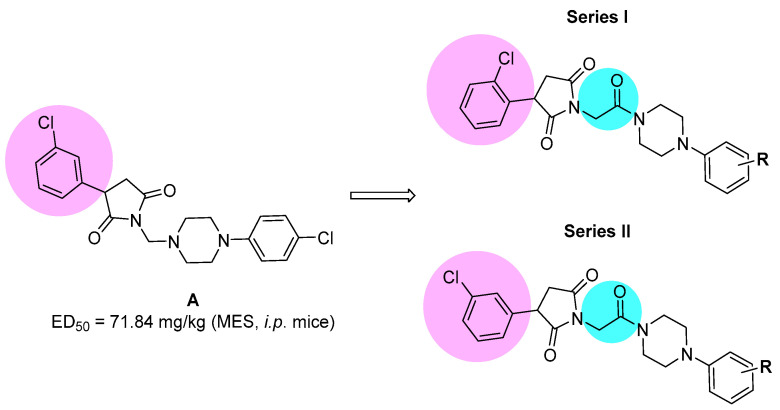
The chemical prototype **A** [8] synthesized previously, and proposed structural modifications for compounds reported in the current studies.

Our previous research showed that the five-membered heterocyclic rings derivatives, i.e., pyrrolidine-2,5-dione, imidazolidine-2,4-dione, pyrrolidin-2-one, revealed anticonvulsant properties in “classical” animal models of seizures, i.e., MES, *sc*PTZ, as well as in the 6 Hz seizure tests [6,7]. In the research conducted so far, we obtained many active compounds among derivatives containing electron-withdrawing atoms/groups in the phenyl substituent at position-3 of pyrrolidine-2,5-dione ring. The potent anticonvulsant activity showed especially compounds that also possess a chlorine atom in the phenyl ring of the arylpiperazine moiety and methylene linker between imide and piperazine fragment (so called N-Mannich bases, see Figure 1, compound **A**) [8]. Notably, many of previously obtained compounds also demonstrated significant analgesic activity in various animal models of pain. The antinociceptive properties were assessed, i.a., in acute (hot plate test) and tonic (formalin test) pain models as well as in the oxaliplatin-induced neuropathic pain model in mice [6].

Taking into consideration the above mentioned facts, we designed and synthesized herein a new series of amide derivatives, which are analogues of the previously obtained and pharmacologically promising N-Mannich bases (see chemical prototype **A**, Figure 1). The modification consisted of replacement of the methylene linker between the imide nitrogen atom and arylpiperazine with acetamide fragment. The main reason for this modification was the increase of chemical and metabolic stability of new compounds (as Mannich bases are sensitive for, e.g., pH and are also rapidly metabolized), and in consequence to assess the influence of introduction of amide function on anticonvulsant properties. Additionally, to evaluate the biological effect of the chlorine substitution at the phenyl ring at the 3-position of pyrrolidine-2,5-dione, we synthesized two series of isomers—namely, with chlorine atom at *ortho* (series I) or *meta* (series II) position (for details see Figure 1). 

The anticonvulsant activity was assessed in acute models of seizures—namely, the MES (maximal electroshock seizure) test and the 6 Hz (psychomotor seizure) test at current intensity of 32 mA. Furthermore, selected substances that were active in the aforementioned electrically induced seizures were also assessed in the *sc*PTZ (subcutaneous pentylenetetrazol) test. The antinociceptive properties were determined in the formalin model of tonic pain. To determine the plausible mechanism of anticonvulsant action for the chosen active compounds, in vitro ion channel binding assays were also carried out. Additionally, for the most promising compounds, hepatotoxic and neurotoxic properties using in vitro cellular models were assessed.

## 2. Results and Discussion

### 2.1. Synthesis and Drug-Likeness Properties 

**Scheme 1:**
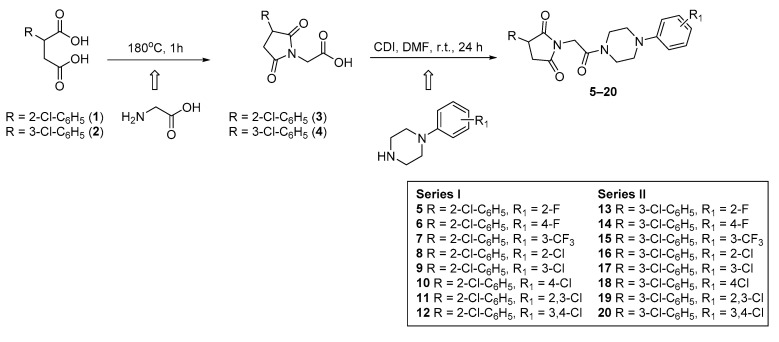
Synthetic procedure of compounds **5**–**20**.

The final compounds **5**–**20** were synthesized according to the procedure depicted in Scheme 1. The starting materials 2-(2-chlorophenyl)-(**1**) and 2-(3-chlorophenyl)-(**2**) succinic acids were prepared in line with the method described by Miller and Long [9]. In the next step, the cyclocondensation of **1** or **2** with aminoacetic acid yielded in 3-(2-chlorophenyl)-(**3**) and 3-(3-chlorophenyl)-2,5-dioxo-pyrrolidin-1-yl-(**4**) acetic acids. The final compounds **5**–**20** were obtained in the coupling reaction of intermediates **3** (series I) or **4** (series II) with equimolar amounts of appropriate 4-arylpiperazines in the presence of carbonyldiimidazole (CDI). The reaction was carried out in dry N,N-dimethylformamide (DMF) as solvent at room temperature for 24 h. The crude products were crystallized from 2-propanol. Compounds **5**–**20** were obtained as a racemic mixtures with yield ranging between 22% and 45%. Their purity and homogeneity were assessed by thin layer chromatography (TLC) and gradient high-performance liquid chromatography (HPLC). The chemical structures were confirmed by spectral analyses (^1^HNMR, ^13^CNMR, LC/MS). The detailed physical and analytical data are listed in the Materials and Methods section.

In the next step, the physicochemical properties of the final compounds were determined based on Lipinski and Veber rules using an online tool—SwissAdme website [10] (Table 1).

The Lipinski and Veber rules are used to assess drug-likeness properties and indicates whether the compound may be a candidate for an orally active drug in humans. Compounds that do not comply with at least two of the criteria of the Lipinski rules may have problems with bioavailability from the gastrointestinal track. The criteria of Lipinski rule are: molecular weight (MW) ≤ 500 Da, lipophilicity value (log*p*) ≤ 5, number of hydrogen bond donors (NHD) ≤ 5, number of hydrogen bond acceptors (NHA) ≤ 10. The Veber rule includes: rotatable bonds (NBR) ≤ 10 and topological polar surface area (TPSA) ≤ 140 Å^2^ [11,12]. 

All tested compounds complied with Lipinski and Veber rules, as they possess fewer than 5 HBD, fewer than 10 HBA, MW below 500 Da, log*p* value < 5, NBR fewer than 10 and PSA value lower than 140 Å^2^. 

**Figure 2:**
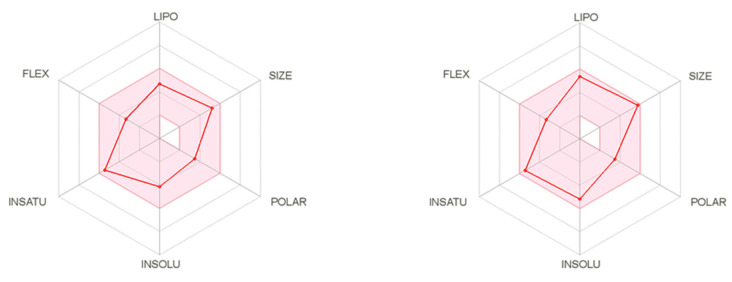
The bioavailability radar of compounds **6** (**left**) and **19** (**right**).

The SwissAdme website also provides radar charts (Figure 2) showing the relationship between oral bioavailability and chemical structure. It considers six physicochemical properties, namely lipophilicity, size, polarity, solubility, flexibility, and saturation of the molecule. These physicochemical properties for two the most active compounds **6** and **19**, are displayed as pink dots, while the pink area represented an acceptable range of physicochemical parameters according to Lipinski and Veber rules (Figure 2). Thus, it can be concluded that these compounds meet the drug-likeness requirements according to Lipinski and Veber rules. Although Lipinski and Veber rules are guidelines for structural drug-likeness properties of compounds, recent research among existing drugs and drug candidates showed that many oral medications are found far beyond Lipinski rule; therefore, compounds which did not comply with all Veber or Lipinski rules should not be disqualified as a promising candidates for an effective drugs [13,14]. 

### 2.2. Anticonvulsant Activity

Anticonvulsant screening of all final compounds was performed initially in the MES and the 6 Hz (32 mA) seizure tests at a fixed dose of 100 mg/kg, 0.5 h after intraperitoneal (i.p.) injection. Each screening group consisted of four animals. All compounds were evaluated as free bases.

The preliminary pharmacological results showed that compounds **5**, **6**, **10**, **13**, **14**, **17**, and **18** revealed in general weak anticonvulsant activity in the MES test, protecting at least 25% of mice (Table 2). The highest and significant anticonvulsant activity in this test was displayed by **6**, which provides 100% protection (4/4) of animals from seizures. In general, more potent activity was observed in the 6 Hz test (32 mA), as eight compounds showed anticonvulsant activity—namely, **5–7**, **10**, **11**, **14**, **15**, and **19**. Notably, half of them (**6**, **7**, **15**, **19**) exhibited significant (at least of 50%) anticonvulsant protection. The highest activity, similar to MES seizures, revealed compound **6**, which protected 75% (3/4) of tested animals. Three other compounds, **7**, **15**, and **19**, were slightly less effective and demonstrated 50% protection. In the rotarod test (NT column, Table 2), which is a measure of acute neurological toxicity, all tested compounds were devoid or revealed negligible neurotoxicity (Table 2). 

Next, the most active compound **6** was initially assessed in the *sc*PTZ test at a fixed dose of 100 mg/kg after i.p. injection at a time point of 0.5 h. In this seizure model, **6** provided 50% (2/4) protection of the tested animals. Surprisingly, at a higher dose of 130 mg/kg, compound **6** protected only 16.7% (1/6) of tested animals, thus the median effective dose (ED_50_) was not determined in the *sc*PTZ test. For details, see Figures S1 and S2 in Supplementary Materials.

In the next step of pharmacological characterization for the most active compounds **6** and **19** selected in screening studies, the ED_50_ values were evaluated in the MES and 6 Hz tests, and the median neurotoxic doses (TD_50_) were determined in the rotarod test. Based on these data, the protective indexes (PIs) were calculated. The PIs values describet the benefit–risk ratio of the therapeutic agent. The results, along with data for reference drugs such as ethosuximide (ETX) and valproic acid (VPA), are summarized in Table 3. 

As shown in Table 3, compound **6** exhibited about c.a. 3-fold more beneficial ED_50_ and PI value than VPA in the MES test and almost 5-fold better ED_50_ value and c.a. 3-fold better PI value than VPA in the 6 Hz test. This compound (**6**) also exhibited 6-fold lower ED_50_ value and 4-fold better PI value than ETX in the 6 Hz test. Compound **19** showed weaker activity, nevertheless slightly better than VPA and ETX in the 6 Hz test.

### 2.3. Antinociceptive Activity in the Formalin Test

The formalin test, a model of both acute chemical pain (acute phase) and tonic nociception involving central sensitization (late phase), is considered the as most usefull model for screening of new compounds with antinociceptive activity because it is the one that most resembles clinical pain compared to other animal pain tests [16]. In mice, intraplantarly injection of diluted formalin produces a biphasic nocifensive behavioral response (i.e., licking or biting the injected hind paw). The acute nociceptive phase lasts for the first 5 min and is directly related to the stimulation of primary sensory neurons (neurogenic pain), whereas the second inflammatory phase occurs between 15 and 30 min after formalin injection, which is associated with inflammation resulting from release of pro-inflammatory mediators, such as adenosine, bradykinin, histamine, prostaglandin, and serotonin [17,18,19]. It has been also reported that the late phase is considered to be a model of central sensitization of pain, which is characterized by pain-induced functional changes in the dorsal horn of the spinal cord. These phenomena are responsible for neuroplasticity of the central nervous system and the development of chronic neuropathic pain, as well as its resistance to analgesic drugs [17]. The results from pain studies with AEDs suggests that these drugs have little or no effect on most measures of normal transient nociceptive signaling but rather inhibit sensitized signaling associated with allodynia (experiencing pain from a stimulus that does not normally trigger a pain response) and hyperalgesia (experiencing increased pain from a stimulus that is normally perceived as less painful), the symptoms of neuropathic pain [18]. Thus, AEDs as well as antidepressants have been the most studied drugs in neuropathic pain mangment [20]. Numerous preclinical studies have demonstrated strong analgesic activity of anticonvulsants (e.g., tiagabine, lamotrigine, gabapentin, and lacosamide) in a wide panel of animal models of pain, including the formalin test [17,21]. In this test, AEDs are active in both phases of the test or only in the second (inflammatory) phase. In our previous experiments, valproic acid significantly decreased the duration time of the licking/biting responses at the doses of 150 mg/kg and 200 mg/kg in both phases, whereas pregabalin tested at the doses of 1–30 mg/kg revealed an antinociceptive effect in the second phase of the formalin test [22,23].

**Figure 3:**
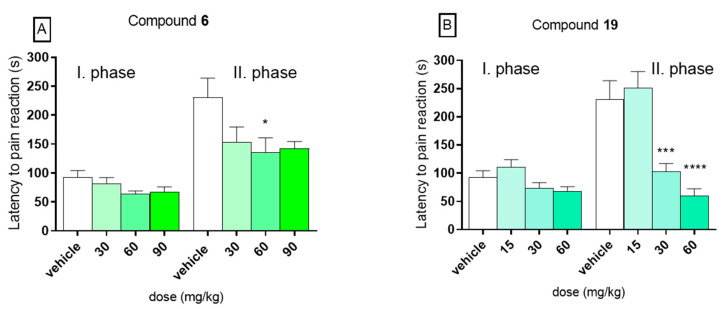
Influence of compounds **6** (panel **A**) and **19** (panel **B**) on duration of licking/biting behavior in the first (neurogenic) phase (0–5 min after formalin injection) and in the second (inflammatory) phase (15–30 min after formalin injection) of the formalin test in mice. Data are presented as mean ± SEM. Statistical analysis: one-way analysis of variance (ANOVA), followed by Dunnett’s test: * *p* < 0.05, *** *p* < 0.001, **** *p* < 0.0001, *n* = 8.

In the present experiment, in the control group the mean time spent on licking or biting the hind paw was 92.1 s ± 12.3 in the first phase and 230.5 s ± 33.5 in the second phase. In the first phase of the formalin test, no statistically significant activity was observed for both compounds **6** (panel A) and **19** (panel B). In this phase, compound **6** at doses 30, 60, and 90 mg/kg diminished the duration time of the licking/biting responses to 81.6 s ± 10.5, 63.5 s ± 5.5, or 67.5 s ± 8.6, respectively. The results recorded for **19**, tested at doses 15, 30, and 60 mg/kg, were 110.3 s ± 13.6, 73.9 s ± 9.3, and 67.7 s ± 8.4, respectively. On the other hand, significant analgesic activity was observed in the second (late) phase of the formalin test for compound **19** at doses 30 and 60 mg/kg. At dose of 30 mg/kg, it significantly decreased the duration of the pain response to 102.9 s ± 14.2 (by 55%, *p* < 0.001) and at dose of 60 mg/kg to 59.9 s ± 12.5 (by 74%, *p* < 0.0001). No analgesic activity was observed at a dose of 15 mg/kg. In the second, phase compound **6**, tested at doses 30, 60, and 90 mg/kg diminished the duration time of the licking/biting responses to 153.3 s ± 26.3 (by 33%), 135.0 s ± 25.8 (by 41%), and 142.4 s ± 12.3 (by 38%), but it reduced the perception of pain in a statistically significant way (*p* < 0.05) only at dose of 60 mg/kg. The results are presented in Figure 3. The analgesic activity in the second phase of this model suggests an anti-inflammatory profile of tested compounds, as well as the ability to inhibit the development of central sensitization of pain. Such properties of these compounds might be of a great therapeutic value in terms of their possible use in treatment of the neuropathic pain. Such compounds with anticonvulsant and collateral analgesic activity can be promising lead structures in the search for novel analgesic adjuvants.

### 2.4. In Vitro Radioligand Binding Studies

Considering the fact that the mechanism of action of many antiepileptic/antinociceptive drugs is often connected with their influence on sodium and/or calcium channels and similar interactions published previously for our compounds [6,7,8,9,10,11,12], for the most active compounds **6** and **19** the binding assays to these ion channels was performed.

As indicated in Table 4, compound **6** exhibited the highest affinity to the voltage-sensitive Na^+^ channel-site 2 (80.0%) and voltage-sensitive Ca^2+^ channel L-type (82.9%) at concentration of 100 μM, whereas compound **19** revealed relatively high interaction with voltage-sensitive Na^+^ channel (site 2) (73.1%) but weak to voltage-sensitive Ca^2+^ channel L-type (35.5%) at concentration of 100 μM. 

### 2.5. Hepatotoxicity and Neurotoxicity Study

**Figure 4:**
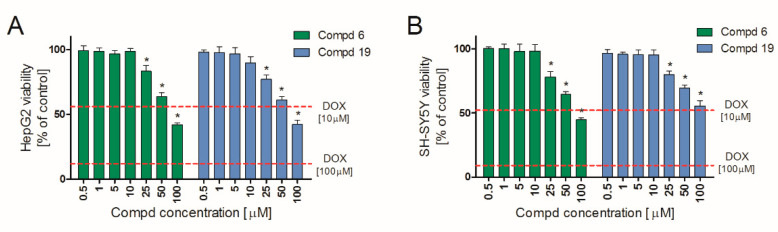
HepG2 (**A**) and SH-SY5Y (**B**) cell lines viability in the presence of **6** and **19**. Cells were cultured with tested compounds for 24 h and then MTT assay was performed. Each bar represents viability expressed as mean % of control (non-treated cells) ± SEM. The dashed lines indicate the toxicity of the positive control (doxorubicin, DOX). * Statistical significance versus control (*p* < 0.05).

Hepatotoxicity and neurotoxicity are the most important reasons for failure of compounds at various stages of drug development. Early in vitro tests minimize the risk of later exclusion of potential drug candidates due to organ toxicity. Therefore, we determined in vitro hepatotoxic and neurotoxic effects of **6** and **19** in two widely approved [24,25] cell lines: HepG2 and SHSY, respectively. The obtained results indicate that both tested compounds are characterized by a similar safety profile. They showed no significant hepatotoxic (Figure 4A) and neurotoxic (Figure 4B) effects in vitro up to 10 μM. Importantly, at concentration range of 0.5–10 μM, they did not affect the viability of either HepG2 or SH-SY5Y cell lines. A slight decrease in viability was observed only at concentration 25 µM and higher. Simultaneously, doxorubicin (DOX), used as a positive control, applied already at 10 µM, showed both hepatotoxic and neurotoxic effects, reducing cell viability by almost 50%, against control. Therefore, **6** and **19** may be considered safe in these in vitro preliminary studies. 

## 3. Materials and Methods 

### 3.1. Chemistry

#### 3.1.1. General Remarks

All the chemicals and solvents were purchased from Sigma-Aldrich (St. Louis, MO, USA) and were used without further purification. Melting points (m.p.) were determined in open capillaries on a Büchi 353 melting point apparatus (Büchi Labortechnik, Flawil, Switzerland). The purity and homogeneity of the compounds were confirmed by thin layer chromatography (TLC) and UPLC gradient chromatography. The TLC was performed on Merck silica gel 60 F_254_ aluminum sheets (Merck, Darmstadt, Germany). Spots were detected by their absorption under UV light (λ = 254 nm). The UPLC analyses and mass spectra (LC-MS) were obtained on Waters ACQUITY TQD system with the MS-TQ detector and UV−vis−DAD eλ detector (Waters, Milford, CT, USA). The ACQUITY UPLC BEH C18, 1.7 μm (2.1 mm × 100 mm) column was used with the VanGuard Acquity UPLC BEH C18, 1.7 μm (2.1 mm × 5 mm) (Waters, Milford, CT, USA). Standard solutions (1 mg/mL) were prepared in analytical grade MeCN/water mixture (1:1; *v*/*v*). Conditions applied were as follows: eluent A (water/0.1% HCOOH), eluent B (MeCN/0.1% HCOOH), a flow rate of 0.3 mL/min, a gradient of 5–100% B over 10 min, and an injection volume of 10 μL. The UPLC retention times (t_R_) are given in minutes. The purity of all intermediates and final compounds assessed by use of UPLC chromatography was >95%. Column chromatography was performed using silica gel 60 (particle size 0.063–0.200; 70–230 Mesh ATM) purchased from Merck. ^1^H-NMR and ^13^C-NMR spectra were obtained in a Varian Mercury spectrometer (Varian Inc., Palo Alto, CA, USA) in CDCl_3_ or DMSO operating in 300 MHz and JEOL (JNM-ECZR500 RS1 ECZR) apparatus operating at 500 MHz using a solvent as an internal standard. Chemical shifts are reported in δ values (ppm), the *J*-values are expressed in hertz (Hz). Signal multiplicities are represented by the following abbreviations: s (singlet), brs (broad singlet), d (doublet), t (triplet), dd (double doublet), dt (doublet of triplets), td (triplet of doublets), and m (multiplet).

The 2-(2-chlorophenyl)-(**1**) and 2-(3-chlorophenyl)-succinic (**2**) acids were prepared in line with the method described by Miller and Long [9]. The physicochemical and spectral data are published elsewhere [8].

#### 3.1.2. Chemical Synthesis

##### General Procedure for the Preparation of the 3-(2-chlorophenyl)-(**3**) and 3-(3-chlorophenyl)-2,5-dioxo-pyrrolidin-1-yl-acetic Acids (**4**)

(*R,S*)-2-(2-Chlorophenyl)- or (*R,S*)-2-(3-chlorophenyl)-succinic acids **(1**, **2**) (0.04 mol) were dissolved in 20 mL of water, and 2-aminoacetic acid (0.04 mol) was gradually added. The mixtures were heated in a term-regulated sand bath with simultaneous distillation of water. After complete removal of water, the temperature of the reaction mixture raised up to 180 °C and was maintained for ca. 1.5 h. The crude products, (*R,S*)-3-(2-chlorophenyl)-2,5-dioxo-pyrrolidin-1-yl-acetic acid (**3**) and (*R,S*)-3-(3-chlorophenyl)-2,5-dioxo-pyrrolidin-1-yl-acetic acid (**4**) were recrystallized from methanol.

*3-(2-Chlorophenyl)-pyrrolidin-2,5-dione-acetic acid (***3***).* Yellow oil. Yield: 75.0%; TLC: *R*_f_ = 0.43 (S_1_); UPLC: *t*_R_ = 4.38 min; ^1^H-NMR (300 MHz, CDCl_3_): δ ppm 2.76 (dd, 1H, CH_2_, *J* = 18.4, 5.5 Hz), 2.90–3.12 (m, 1H, CH_2_), 3.25–3.39 (m, 1H, CH), 4.28–4.33 (m, 2H, CH_2_), 6.64 (br. s., 1H, COOH), 7.13–7.31 (m, 3H, ArH), 7.34–7.43 (m, 1H, ArH); ^13^C-NMR (75 MHz, CDCl_3_): δ ppm 35.27, 39.06, 44.52, 127.25, 129.39, 129.82, 130.08, 133.48, 134.98, 170.86, 175.37, 176.90; C_12_H_10_ClNO_4_ (267.67); monoisotopic mass 267.03; [M + H]^+^ = 266.0, 268.0.

*3-(3-Chlorophenyl)-pyrrolidin-2,5-dione-acetic acid (***4***).* Yellow oil. Yield: 64.0%; TLC: *R*_f_ = 0.45 (S_1_); UPLC: *t*_R_ = 4.85 min; ^1^H-NMR (300 MHz, CDCl_3_): δ ppm 2.75–2.81 (m, 1H, CH_2_), 2.92–3.15 (m, 1H, CH_2_), 3.20–3.35 (m, 1H, CH_2_), 4.25–4.30 (m, 2H, CH_2_), 7.10–7.45 (m, 4H, ArH); ^13^C-NMR (75 MHz, CDCl_3_): δ ppm 36.18, 39.45, 44.72, 127.21, 129.41, 129.89, 130.92, 131.52, 134.98, 171.96, 176.97, 177.92; C_12_H_10_ClNO_4_ (267.67); monoisotopic mass 267.03; [M + H]^+^ = 266.4, 267.9.

##### General Procedure for the Synthesis of Compounds **5**–**20**

The obtained intermediates (**3**, **4**) (0.01 mol) were dissolved in 20 mL of DMF and N,N-carbonyldiimidazole (0.01 mol) was added. The mixtures were stirred for 0.5 h at a room temperature. Afterward, the appropriate 4-arylpiperazine (0.01 mol) dissolved in 5 mL of DMF was added. After 24 h of stirring the reaction mixtures were left in an ice-cold bath. The products were precipitated out with cold water and were purified by recrystallization from isopropyl alcohol, giving the final compounds as solids in yields ranging from 22% to 45.5%.

*(R,S)-3-(2-Chlorophenyl)-1-{2-[4-(2-fluorophenyl)piperazin-1-yl]-2-oxoethyl}-pyrrolidine-2,5-dione(***5***).* White powdery crystals. Yield: 34.8%; m.p. 125.0–127.0 °C; TLC: *R*_f_ = 0.63 (S_1_); UPLC: *t*_R_ = 6.75 min; ^1^H-NMR (300 MHz, CDCl_3_): δ ppm 2.79 (dd, 1H, CH_2_, *J* = 18.2, 5.3 Hz), 3.04–3.19 (m, 4H, piperazine), 3.36 (dd, 1H, CH_2_, *J* = 18.5, 9.7 Hz), 3.62–3.85 (m, 4H, piperazine), 4.46 (s, 2H, CH_2_), 4.56 (dd, 1H, CH, *J* = 9.7, 5.6 Hz), 6.89–7.13 (m, 4H, ArH), 7.20–7.34 (m, 2H, ArH), 7.35–7.44 (m, 2H, ArH); ^13^C-NMR (75 MHz, CDCl_3_): δ ppm 36.91, 39.96, 42.43, 44.23, 44.97, 50.14, 50.58, 116.16, 119.17, 123.40, 124.45, 127.82, 129.32, 129.67, 133.79, 135.47, 139.32, 154.10, 157.37, 163.20, 175.38, 177.01; C_22_H_21_N_3_O_3_ClF (429.88); monoisotopic mass 429.13; [M + H]^+^ = 430.2, 432.1.

*(R,S)-3-(2-Chlorophenyl)-1-{2-[4-(4-fluorophenyl)piperazin-1-yl]-2-oxoethyl}-pyrrolidine-2,5-dione (***6***).* White powdery crystals. Yield: 21.1%; m.p. 112.0–114.0 °C; TLC: *R*_f_ = 0.50 (S_1_); UPLC: *t*_R_ = 6.57 min; ^1^H-NMR (300 MHz, CDCl_3_): δ ppm 2.71–2.87 (m, 1H, CH_2_), 3.12 (dt, 4H, piperazine, *J* = 19.8, 5.1 Hz), 3.35 (dd, 1H, CH_2_, *J* = 18.5, 9.7 Hz), 3.61–3.83 (m, 4H, piperazine), 4.45 (s, 2H, CH_2_), 4.55 (dd, 1H, CH, *J* = 9.9, 5.3 Hz), 6.84–7.04 (m, 4H, ArH), 7.22–7.45 (m, 4H, ArH); ^13^C-NMR (75 MHz, CDCl_3_): δ ppm 36.89, 39.96, 42.31, 44.25, 44.78, 50.31, 50.52, 115.61, 115.92, 118.85, 127.00, 128.22, 128.92, 130.29, 133.79, 135.44, 147.43, 156.16, 159.35, 163.23, 175.36, 177.01; C_22_H_21_N_3_O_3_ClF (429.88); monoisotopic mass 429.13; [M + H]^+^ = 430.2, 432.2.

*(R,S)-3-(2-Chlorophenyl)-1-{2-[4-(3-trifluoromethylphenyl)piperazin-1-yl]-2-oxoethyl}-pyrrolidine-2,5-dione (***7***).* White powdery crystals. Yield: 39.0%; m.p. 106.0–108.0 °C; TLC: *R*_f_ = 0.66 (S_1_); UPLC: *t*_R_ = 7.41 min; ^1^H-NMR (300 MHz, CDCl_3_): δ ppm 2.71–2.87 (m, 1H, CH_2_), 3.19–3.35 (m, 4H, piperazine), 3.35–3.51 (m, 1H, CH_2_), 3.64–3.86 (m, 4H, piperazine), 4.46 (s, 2H, CH_2_), 4.56 (dd, 1H, CH, *J* = 9.7, 5.6 Hz), 7.03–7.19 (m, 3H, ArH), 7.21–7.45 (m, 5H, ArH); ^13^C-NMR (75 MHz, CDCl_3_): δ ppm 36.88, 39.93, 42.04, 44.28, 44.52, 48.78, 48.90, 112.60, 113.39, 117.01, 119.47, 122.33, 127.81, 129.35, 129.46, 130.2, 133.77, 135.38, 150.39, 151.53, 163.33, 175.36, 176.99; C_23_H_21_N_3_O_3_F_3_Cl (479.88); monoisotopic mass 479.12; [M + H]^+^ = 480.2, 482.2.

*(R,S)-3-(2-Chlorophenyl)-1-{2-[4-(2-chlorophenyl)piperazin-1-yl]-2-oxoethyl}-pyrrolidine-2,5-dione (***8***).* White powdery crystals. Yield: 30.0%; m.p. 85.0–87.5 °C; TLC: *R*_f_ = 0.63 (S_1_), UPLC: *t*_R_ = 7.22 min; ^1^H-NMR (300 MHz, CDCl_3_): δ ppm 2.79 (dd, 1H, CH_2_, *J* = 18.12, 5.3 Hz), 3.09 (dt, 4H, piperazine, *J* = 19.8, 4.8 Hz), 3.36 (dd, 1H, CH_2_, *J* = 18.5, 9.7 Hz), 3.64–3.85 (m, 4H, piperazine), 4.46 (s, 2H, CH_2_), 4.56 (dd, 1H, CH, *J* = 9.4, 5.3 Hz), 6.96–7.07 (m, 2H, ArH), 7.19–7.33 (m, 3H, ArH), 7.34–7.44 (m, 3H, ArH); ^13^C-NMR (75 MHz, CDCl_3_): δ ppm 36.92, 40.02, 42.63, 44.23, 45.12, 50.81, 51.21, 120.63, 124.49, 127.76, 127.82, 128.97, 129.17, 129.65, 129.95, 130.74, 133.80, 135.48, 148.41, 163.27, 175.38, 177.02; C_22_H_21_N_3_O_3_Cl_2_ (446.33); monoisotopic mass 445.10; [M + H]^+^ = 446.3, 448.3, 450.3.

*(R,S)-3-(2-Chlorophenyl)-1-{2-[4-(3-chlorophenyl)piperazin-1-yl]-2-oxoethyl}-pyrrolidine-2,5-dione (***9***).* White powdery crystals. Yield: 25.5%; m.p. 119–121 °C; TLC: *R*_f_ = 0.71 (S_1_); UPLC: *t*_R_ = 7.20 min; ^1^H-NMR (300 MHz, CDCl_3_): δ ppm 2.79 (dd, 1H, CH_2_, *J* = 18.5, 5.6 Hz), 3.23 (dt, 4H, piperazine, *J* = 18.8, 4.9 Hz), 3.30–3.43 (m, 1H, CH_2_), 3.61–3.82 (m, 4H, piperazine), 4.45 (s, 2H, CH_2_), 4.56 (dd, 1H, CH, *J* = 9.7, 5.6 Hz), 6.75–6.82 (m, 1H, ArH), 6.84–6.92 (m, 2H, ArH), 7.15–7.33 (m, 3H, ArH), 7.35–7.44 (m, 2H, ArH); ^13^C-NMR (75 MHz, CDCl_3_): δ ppm 36.89, 39.95, 42.04, 44.28, 44.52, 48.77, 48.90, 114.59, 116.59, 120.43, 127.82, 129.35, 129.67, 130.25, 129.87, 133.79, 134.96, 135.55, 151.72, 163.29, 175.16, 176.98; C_22_H_21_N_3_O_3_Cl_2_ (446.33); monoisotopic mass 445.10; [M + H]^+^ = 446.1, 448.1, 450.1.

*(R,S)-3-(2-Chlorophenyl)-1-{2-[4-(4-chlorophenyl)piperazin-1-yl]-2-oxoethyl}-pyrrolidine-2,5-dione (***10***).* White powdery crystals. Yield: 22.0%; m.p. 147.0–149.0 °C; TLC: *R*_f_ = 0.58 (S_1_); UPLC: *t*_R_ = 7.15 min; ^1^H-NMR (500 MHz, CDCl_3_): δ ppm 2.78 (dd, 1H, CH_2_, *J* = 18.5, 5.5 Hz), 3.14 (t, 2H, piperazine, *J* = 5.1 Hz), 3.18–3.26 (m, 2H, piperazine), 3.34 (dd, 1H, CH_2_, *J* = 18.5, 9.8 Hz), 3.63–3.71 (m, 2H, piperazine), 3.74–3.84 (m, 2H, piperazine), 4.44 (s, 2H, CH_2_), 4.54 (dd, 1H, CH, *J* = 9.7, 5.5 Hz), 6.86 (d, 2H, ArH, *J* = 8.7 Hz), 7.19–7.32 (m, 4H, ArH), 7.35–7.46 (m, 2H, ArH); ^13^C-NMR (126 MHz, CDCl_3_): δ ppm 36.99, 40.02, 42.16, 44.36, 44.66, 49.50, 49.69, 118.28, 127.92, 129.31, 129.45, 129.76, 130.03, 133.89, 135.49, 163.35, 175.45, 177.09; C_22_H_21_N_3_O_3_Cl_2_ (446.33); monoisotopic mass 445.10; [M + H]^+^ = 446.2, 448.1, 450.1.

*(R,S)-3-(2-Chlorophenyl)-1-{2-[4-(2,3-dichlorophenyl)piperazin-1-yl]-2-oxoethyl}-pyrrolidine-2,5-dione (***11***).* White powdery crystals. Yield: 22.4%; m.p. 160.0–161.5 °C; TLC: *R*_f_ = 0.66 (S_1_); UPLC: *t*_R_ = 7.71 min; ^1^H-NMR (300 MHz, CDCl_3_): δ ppm 2.79 (dd, 1H, CH_2_, *J* = 18.5, 5.6 Hz), 3.07 (dt, 4H, piperazine, *J* = 18.9, 4.9 Hz), 3.36 (dd, 1H, CH_2_, *J* = 18.5, 9.7 Hz), 3.64–3.87 (m, 4H, piperazine), 4.46 (s, 2H, CH_2_), 4.56 (dd, 1H, CH, *J* = 9.7, 5.6 Hz), 6.94 (dd, 1H, ArH, *J* = 7.3, 2.1 Hz), 7.12–7.34 (m, 4H, ArH), 7.35–7.44 (m, 2H, ArH); ^13^C-NMR (75 MHz, CDCl_3_): δ ppm 36.91, 39.99, 42.57, 44.23, 45.06, 50.90, 51.28, 118.86, 125.41, 127.64, 127.82, 129.33. 129.65, 129.91, 133.79, 134.20, 135.44, 150.33, 163.30, 175.38, 176.59; C_22_H_20_N_3_O_3_Cl_3_ (480.77); monoisotopic mass 479.06; [M + H]^+^ = 480.1, 482.0, 484.2.

*(R,S)-3-(2-Chlorophenyl)-1-{2-[4-(3,4-dichlorophenyl)piperazin-1-yl]-2-oxoethyl}-pyrrolidine-2,5-dione (***12***).* White powdery crystals. Yield: 26.1%; m.p. 134.0–136.0 °C; TLC: *R*_f_ = 0.48 (S_1_); UPLC: *t*_R_ = 7.66 min; ^1^H-NMR (300 MHz, CDCl_3_): δ ppm 2.79 (dd, 1H, CH_2_, *J* = 18.5, 5.6 Hz), 3.19 (dt, 4H, piperazine, *J* = 18.9, 5.2 Hz), 3.35 (dd, 1H, CH_2_, *J* = 18.8, 9.9 Hz), 3.60–3.82 (m, 4H, piperazine), 4.44 (s, 2H, CH_2_), 4.55 (dd, 1H, CH, *J* = 9.9, 5.3 Hz), 6.74 (dd, 1H, ArH, *J* = 8.8, 2.9 Hz), 6.96 (d, 1H, ArH, *J* = 2.9 Hz), 7.21–7.43 (m, 5H, ArH); ^13^C-NMR (75 MHz, CDCl_3_): δ ppm 36.88, 39.92, 41.93, 44.26, 44.42, 48.74, 48.87, 115.99, 118.05, 123.41, 127.81, 129.18, 129.67, 129.94, 130.64, 132.98, 133.77, 135.38, 150.04, 163.32, 175.31, 176.96; C_22_H_20_N_3_O_3_Cl_3_ (480.77); monoisotopic mass 479.06; [M + H]^+^ = 480.1, 482.0, 484.2.

*(R,S)-3-(3-Chlorophenyl)-1-{2-[4-(2-fluorophenyl)piperazin-1-yl]-2-oxoethyl}-pyrrolidine-2,5-dione (***13***).* White powdery crystals. Yield: 35.5%; m.p. 119.0–121.0 °C; TLC: *R*_f_ = 0.79 (S_1_); UPLC: *t*_R_ = 7.02 min; ^1^H-NMR (500 MHz, CDCl_3_): δ ppm 2.80–2.90 (m, 1H, CH_2_), 3.07–3.19 (m, 4H, piperazine), 3.25–3.34 (m, 1H, CH_2_), 3.62–3.85 (m, 4H, piperazine), 4.04 –4.12 (m, 1H, CH), 4.39–4.43 (m, 2H, CH_2_), 6.96–7.10 (m, 4H, ArH), 7.22–7.34 (m, 4H, ArH); ^13^C-NMR (126 MHz, CDCl_3_): δ ppm 32, 65, 37.56, 40.10, 42.44, 44.93, 45.91, 116.48 (d, *J* = 23.5 Hz), 119.65, 123.86 (d, *J* = 7.9 Hz), 124.77 (d, *J* = 3.0 Hz), 125.92, 128.27 (d, *J* = 28.9 Hz), 128.21, 130.61, 134.94, 138.98 (d, *J* = 9.7 Hz), 139.17, 153.00 (d, *J* = 239.0 Hz), 163.16, 175.09, 177.02; C_22_H_21_N_3_O_3_ClF (429.88); monoisotopic mass 429.13; [M + H]^+^ = 430.1, 432.2.

*(R,S)-3-(3-Chlorophenyl)-1-{2-[4-(4-fluorophenyl)piperazin-1-yl]-2-oxoethyl}-pyrrolidine-2,5-dione (***14***).* White powdery crystals. Yield: 25.5%; m.p. 123.5–125.0 °C; TLC: *R*_f_ = 0.71 (S_1_); UPLC: *t*_R_ = 6.85 min; ^1^H-NMR (500 MHz, CDCl_3_): δ ppm 2.80–2.88 (m, 1H, CH_2_), 3.04–3.17 (m, 4H, piperazine), 3.30 (dd, 1H, CH_2_, *J* = 18.5, 9.7 Hz), 3.61–3.82 (m, 4H, piperazine), 4.09 (dd, *J* = 9.6, 4.9 Hz, 1H, CH), 4.41 (d, 2H, CH_2_, *J* = 1.4 Hz), 6.90 (br. s., 1H, ArH), 6.94–7.02 (m, 2H, ArH), 7.22–7.33 (m, 5H, ArH); ^13^C-NMR (126 MHz, CDCl_3_): δ ppm 37.54, 40.09, 42.36, 44.80, 45.90, 115.91 (d, *J* = 21.7 Hz), 119.06 (d, *J* = 6.6 Hz), 125.94, 128.29 (d, *J* = 1.2 Hz), 130.68, 134.98, 139.36, 158.03 (d, *J* = 238.4 Hz), 163.19, 175.48, 177.03; C_22_H_21_N_3_O_3_ClF (429.88); monoisotopic mass 429.13; [M + H]^+^ = 430.2, 432.1.

*(R,S)-3-(3-Chlorophenyl)-1-{2-[4-(3-trifluoromethylphenyl)piperazin-1-yl]-2-oxoethyl}-pyrrolidine-2,5-dione (***15***).* White powdery crystals. Yield: 39.8%; m.p. 144.0–145.5 °C; TLC: *R*_f_ = 0.79 (S_1_); UPLC: *t*_R_ = 7.55 min; ^1^H-NMR (500 MHz, CDCl_3_): δ ppm 2.86 (dd, 1H, CH_2_, *J* = 18.5, 4.9 Hz), 3.19–3.26 (m, 2H, piperazine), 3.26–3.35 (m, 3H, piperazine, CH_2_), 3.64–3.70 (m, 2H, piperazine), 3.75–3.84 (m, 2H, piperazine), 4.10 (dd, 1H, CH, *J* = 9.7, 4.9 Hz), 4.42 (d, 2H, CH_2_, *J* = 1.6 Hz), 7.08 (dd, 1H, ArH, *J* = 8.3, 2.3 Hz), 7.11–7.18 (m, 2H, ArH), 7.21–7.34 (m, 4H, ArH), 7.38 (t, 1H, ArH, *J* = 7.9 Hz); ^13^C-NMR (126 MHz, CDCl_3_): δ ppm 37.54, 40.0, 42.12, 44.56, 45.91, 49.06, 113.22 (q, *J* = 4.2 Hz), 117.32 (q, *J* = 3.0 Hz), 119.70, 124.20 (q, *J* = 272.2 Hz), 125.92, 128.27, 128.31, 129.93, 130.68, 131.75 (q, *J* = 32.0 Hz,), 135.00, 139.32, 150.77, 163.28, 175.44, 177.00; C_23_H_21_N_3_O_3_ClF_3_ (479.88); monoisotopic mass 479.12; [M + H]^+^ = 480.2, 482.1.

*(R,S)-3-(3-Chlorophenyl)-1-{2-[4-(2-chlorophenyl)piperazin-1-yl]-2-oxoethyl}-pyrrolidine-2,5-dione (***16***).* White powdery crystals. Yield: 45.5%; m.p. 113.5–119.5 °C; TLC: *R*_f_ = 0.84 (S_1_); UPLC: *t*_R_ = 7.49 min; ^1^H-NMR (500 MHz, CDCl_3_): δ ppm 2.85 (dd, 1H, CH_2_, *J* = 18.5, 4.9 Hz), 3.01–3.15 (m, 4H, piperazine), 3.30 (dd, 1H, CH_2_, *J* = 18.5, 9.7 Hz), 3.62–3.85 (m, 4H, piperazine), 4.09 (dd, 1H, CH, *J* = 9.6, 4.9 Hz), 4.41 (d, 2H, CH_2_, *J* = 1.6 Hz), 6.99–7.06 (m, 2H, ArH), 7.20–7.34 (m, 5H, ArH), 7.37 (dd, 1H, ArH, *J* = 7.9, 1.5 Hz); ^13^C-NMR (126 MHz, CDCl_3_): δ ppm 37.53, 40.13, 42.65, 45.13, 45.90, 50.89, 51.30, 120.29, 121.07, 124.52, 125.55, 126.05, 127.61, 127.97, 128.14, 128.75, 130.62, 130.83, 147.86, 163.24, 175.49, 177.03; C_22_H_21_N_3_O_3_Cl_2_ (446.33); monoisotopic mass 445.10; [M + H]^+^ = 446.1, 448.1, 450.1.

*(R,S)-3-(3-Chlorophenyl)-1-{2-[4-(3-chlorophenyl)piperazin-1-yl]-2-oxoethyl}-pyrrolidine-2,5-dione (***17***).* White powdery crystals. Yield: 39.5%; m.p. 154.0–156.0 °C; TLC: *R*_f_ = 0.84 (S_1_); UPLC: *t*_R_ = 7.45 min; ^1^H-NMR (500 MHz, CDCl_3_): δ ppm 2.86 (dd, 1H, CH_2_, *J* = 18.5, 4.9 Hz), 3.21 (t, 2H, piperazine, *J* = 5.0 Hz), 3.26–3.36 (m, 3H, piperazine, CH_2_), 3.66–3.86 (m, 4H, piperazine), 4.10 (dd, 1H, CH, *J* = 9.7, 4.9 Hz), 4.42 (s, 2H, CH_2_), 6.85–6.98 (m, 3H, ArH), 7.18–7.24 (m, 2H, ArH), 7.26–7.34 (m, 3H, ArH); ^13^C-NMR (126 MHz, CDCl_3_): δ ppm 37.54, 40.04, 41.71, 44.23, 45.91, 50.95, 114.98, 116.98, 117.38, 125.91, 128.30, 130.47, 130.69, 134.85, 135.10, 135.20, 135.66, 139.29, 163.41, 175.31, 176.83; C_22_H_21_N_3_O_3_Cl_2_ (446.33); monoisotopic mass 445.10; [M + H]^+^ = 446.1, 448.2. 450.1.

*(R,S)-3-(3-Chlorophenyl)-1-{2-[4-(4-chlorophenyl)piperazin-1-yl]-2-oxoethyl}-pyrrolidine-2,5-dione (***18***).* White powdery crystals. Yield: 39.5%; m.p. 110.0–112.0 °C; TLC: *R*_f_ = 0.76 (S_1_); UPLC: *t*_R_ = 7.42 min; ^1^H-NMR (500 MHz, CDCl_3_): δ ppm 2.85 (dd, 1H, CH_2_, *J* = 18.5, 4.9 Hz), 3.11–3.24 (m, 4H, piperazine), 3.30 (dd, 1H, CH_2_, *J* = 18.5, 9.6 Hz), 3.61–3.81 (m, 4H, piperazine), 4.09 (dd, 1H, CH, *J* = 9.6, 4.9 Hz), 4.41 (d, 2H, CH_2_, *J* = 1.3 Hz), 6.86 (d, 2H, ArH, *J* = 8.7 Hz), 7.20–7.34 (m, 6H, ArH); ^13^C-NMR (126 MHz, CDCl_3_): δ ppm 37.54, 40.07, 42.15, 44.60, 45.90, 49.55, 49.74, 118.33, 125.9, 128.29, 129.32, 130.68, 134.99, 139.3, 163.22, 175.45, 177.00; C_22_H_21_N_3_O_3_Cl_2_ (446.33); monoisotopic mass 445.10; [M + H]^+^ = 446.1, 448.1, 450.3.

*(R,S)-3-(3-Chlorophenyl)-1-{2-[4-(2,3-dichlorophenyl)piperazin-1-yl]-2-oxoethyl}-pyrrolidine-2,5-dione (***19***).* White powdery crystals. Yield: 34.9%; m.p. 120.0–122.0 °C; TLC: *R*_f_ = 0.81 (S_1_); UPLC: *t*_R_ = 7.85 min; ^1^H-NMR (500 MHz, CDCl_3_): δ ppm 2.82–2.89 (m, 1H, CH_2_), 3.00–3.13 (m, 4H, piperazine), 3.30 (dd, 1H, CH_2_, *J* = 18.5, 9.7 Hz), 3.63–3.84 (m, 4H, piperazine), 4.10 (dd, 1H, CH, *J* = 9.6, 4.9 Hz), 4.41 (d, 2H, CH_2_, *J* = 1.7 Hz), 6.90–6.95 (m, 1H, ArH), 7.13–7.34 (m, 6H, ArH); ^13^C-NMR (126 MHz, CDCl_3_): δ ppm 37.55, 40.14, 42.68, 45.13, 45.91, 50.98, 51.38, 118.96, 125.54, 125.93, 127.73, 128.29, 130.68, 134.33, 134.99, 139.36, 150.40, 163.27, 175.48, 177.02; C_22_H_20_N_3_O_3_Cl_3_ (480.77); monoisotopic mass 479.06; [M + H]^+^ = 480.0, 482.3, 484.0.

*(R,S)-3-(3-Chlorophenyl)-1-{2-[4-(3,4-dichlorophenyl)piperazin-1-yl]-2-oxoethyl}-pyrrolidine-2,5-dione (***20***).* White powdery crystals. Yield: 38.5%; m.p. 149.0–150.5 °C; TLC: *R*_f_ = 0.76 (S_1_); UPLC: *t*_R_ = 7.79 min; ^1^H-NMR (500 MHz, CDCl_3_): δ ppm 2.85 (dd, 1H, CH_2_, *J* = 18.5, 4.9 Hz); 3.13–3.25 (m, 4H, piperazine); 3.30 (dd, 1H, CH_2_, *J* = 18.6, 9.6 Hz); 3.60–3.82 (m, 4H, piperazine); 4.09 (dd, 1H, CH, *J* = 9.6, 4.9 Hz), 4.40 (d, 2H, CH_2_, *J* = 1.4 Hz), 6.76 (dd, 1H, ArH, *J* = 8.8, 2.9 Hz), 6.98 (d, 1H, ArH, *J* = 2.7 Hz), 7.20–7.34 (m, 5H, ArH); ^13^C-NMR (126 MHz, CDCl_3_): δ ppm 37.54, 40.05, 41.99, 44.44, 45.90, 49.01, 49.14, 116.25, 118.34, 123.89, 125.92, 128.26, 128.32, 130.68, 130.80, 133.15, 135.00, 139.30, 149.89, 163.27, 175.43, 176.99; C_22_H_20_N_3_O_3_Cl_3_ (480.77); monoisotopic mass 479.06; [M + H]^+^ = 480.2, 482.1, 484.1.

### 3.2. In Vivo Experiments

#### 3.2.1. Animals 

Male CD-1 mice weighing 20–26 g were used in the in vivo experiment. The animals were housed in an environmentally controlled room (temperature of 22 ± 2 °C, humidity 55 ± 10%) on 12 h light/dark cycles (light on at 7:00 AM and off at 7:00 PM) and had free access to food (standard laboratory pellets) and water. The experimental groups consisted of 4–6 mice (anticonvulsant and neurotoxic studies) or 8 animals (antinociceptive studies). The experiments were performed between 8:00 a.m. and 3:00 p.m. For the experiments, the animals were selected in a random way and trained observers performed all measurements. The experimental protocol was approved by the First Local Ethics Committee on Animal Testing at the Jagiellonian University in Kraków (No 131/2017, 159/2018, 365/2020).

#### 3.2.2. Maximal Electroshock Seizure Test (MES)

In the MES test, an electrical stimulus of sufficient intensity (25 mA, 500 V, 50 Hz, 0.2 s) was delivered via auricular electrodes by the electroshock generator (Rodent Shocker, Type 221, Hugo Sachs, March-Hugstetten, Germany) to induce maximal seizures. The endpoint was the tonic extension of the hind limbs. Mice not displaying hind-limb tonic extension were considered to be protected from seizures [26].

#### 3.2.3. The 6 hertz (6 Hz) Psychomotor Seizure Test

In the 6 Hz test, psychomotor seizures were induced via corneal stimulation (6 Hz, 32 mA, 0.2 ms rectangular pulse width, 3 s duration) using a constant-current device (ECT Unit 57800, Ugo Basile, Italy). A drop of 1% solution of lidocaine hydrochloride (Polfa Warszawa, Warsaw, Poland) was applied to the mouse corneas before stimulation to provide local anesthesia and ensure optimal current conductivity. After the electrical stimulation, mice were gently released and observed for the presence or absence of seizure activity, being characterized by immobility associated with rearing, forelimb clonus, twitching of the vibrissae, stun, and Straub-tail (Brown et al. 1953, Barton et al. 2001). Mice resuming normal behavior within 10 s from the stimulation were considered as protected [27,28].

#### 3.2.4. PTZ Seizure Test

In the PTZ seizure test, clonic convulsions were induced by the subcutaneous (*sc*) administration of pentylenetetrazole (Sigma-Aldrich, St. Louis, MO, USA) at a dose of 100 mg/kg in 0.9% saline solution. After PTZ injection, each mouse was placed separately and observed during the next 30 min for the occurrence of clonic seizures, which were defined as clonus of the whole body lasting more than 3 s, with an accompanying loss of righting reflex. Latency time to first clonus episode was noted and compared with the control group. The absence of clonic convulsions within the observed time period was interpreted as the compound’s ability to protect against PTZ-induced seizures [6].

#### 3.2.5. Neurotoxicity Screening (NT)—Rotarod Test

In this test, mice were trained to balance on an accelerating rod that rotated at 10 rotations per minute (Rotarod apparatus, May Commat RR0711, Turkey; rod diameter: 3 cm). During the training session, the animals were placed on a rotating rod for 3 min with an unlimited number of trials. Proper experiment was conducted at least 24 h after the training trial. On the test day, trained mice were intraperitoneally pretreated with the test compound and were evaluated in the rotarod test (at the screening dose of 100 mg/kg just before the MES test or at a dose of 300 mg/kg after 0.5 h). Neurotoxicity was indicated by the inability of the animal to maintain equilibration on the rod for 1 min. 

#### 3.2.6. Median Effective Dose (ED_50_), Median Toxic Dose (TD_50_), and Protective Index (PI)

The ED_50_ is defined as the dose of a drug protecting 50% of animals against the MES and 6 Hz seizure episodes. The neurotoxic effect was expressed as a TD_50_ value, representing the doses at which the compound resulted in minimal motor impairment in 50% of the animals in the rotarod test. To evaluate the ED_50_ or TD_50_, three groups of animals were injected with various doses of tested compounds. Each group consisted of six animals. Both ED_50_ and TD_50_ values with 95% confidence limits were calculated by probit analysis (Litchfield and Wilcoxon, 1949). The PI value was calculated as the ratio of TD_50_ to the respective ED_50_ value, as determined in the MES or 6 Hz tests. The PI is considered as an index of the margin of safety and tolerability between anticonvulsant doses and doses of the compounds exerting acute adverse effects [29].

#### 3.2.7. Formalin Test

Antinociceptive activity in the formalin test was examined according to Laughlin et al. [17] with some minor modification as previously described [23]. The mice were pretreated i.p. with the test compound or vehicle 30 min before the experiment. Subsequently, 20 μL of a 2.5% formalin solution (Sigma-Aldrich, St. Louis, MO, USA) was injected intraplantarly (i.pl.) into the right hind paw of a mouse. Immediately after formalin injection, the animals were placed individually into glass beakers and were observed for the next 30 min. Time (in seconds) spent on licking or biting the injected hind paw in selected intervals, 0–5 and 15–30 min, was measured in each experimental group and was an indicator of nociceptive behavior. 

Results were statistically evaluated using one-way ANOVA, followed by Dunnett’s multiple comparison test. A *p* < 0.05 was considered significant.

### 3.3. In Vitro Experiments

#### 3.3.1. Binding and Functional Assays

The radioligand binding studies were performed commercially by Eurofins Cerep SA (Celle l’Evescault, France) using testing procedures described elsewhere—sodium channels (site 2), L-type calcium channels (dihydropyridine site) [30,31]. Compound binding data were expressed as a percentage of inhibition of the binding/response of a radioactively labelled ligand. All experiments were performed in duplicate.

#### 3.3.2. In Vitro Hepatotoxicity and Neurotoxicity Assessment

To evaluate compounds **6** and **19** neurotoxic or hepatotoxic effects in vitro, a MTT (3-(4,5-dimethylthiazol-2-yl)-2,5diphenyltetrazolium bromide) (Sigma-Aldrich, St. Louis, MO, USA) viability assay was performed. A human neuroblastoma cell line: SH-SY5Y (ATCC^®^, CRL-2266™) and a human hepatocellular carcinoma cell line: HepG2 (ATCC^®^ HB-8065™) were used in the study. Both cell lines were cultured in Dulbecco′s modified Eagle′s medium (DMEM; Gibco, Thermo Scientific, Waltham, MA, USA) supplemented with 10% (*v*/*v*) fetal bovine serum (FBS; Gibco, Thermo Scientific, Waltham, MA, USA) and antibiotics mixture (penicillin, streptomycin, amphotericin B; Gibco, Thermo Scientific, Waltham, MA, USA) in standard culture conditions (5% CO_2_, 37 °C, 95% humidity). Twenty-four hours after seeding, cells were incubated in the presence of 6 and 19 administrated at growing concentrations (0.5–100 µM). The MTT reagent (final concentration 0.5 mg/mL) was added to the culture medium for the last 4 h of 24 h incubation. Then the medium was removed and the formed formazan crystals were dissolved in DMSO (Sigma-Aldrich, St. Louis, MO, USA). Next, the absorbance at 570 nm was measured using a microplate reader (SpectraMax^®^ iD3, Molecular Devices, San Jose, CA, USA). The obtained values were proportional to the number of actively metabolizing cells in the population. The experiment was performed three times in duplicate. Each bar represents mean (±SEM) percentage of viable cells in comparison to control (defined as 100% and represented cells untreated with any compound).

## 4. Conclusions

In the present study, the library of new 3-(2-chlorophenyl)- and 3-(3-chlorophenyl)-pyrrolidine-2,5-dione-acetamides as potential anticonvulsant and antinociceptive agents was synthesized. The obtained results revealed that several compounds exhibited anticonvulsant activity, and none of them showed significant acute neurological toxicity. Among all tested compounds, the most potent and the broadest spectrum of activity was displayed by **6**, with ED_50_ (MES) = 68.30 mg/kg and ED_50_ (6 Hz) = 28.20 mg/kg. Notably, this compound was more effective in given seizure tests and had more beneficial protective index than well-known wide spectrum AED—valproic acid. Furthermore, compound **6** and its dichloro- analogue **19** revealed significant antinociceptive activity in the formalin test, which is a model of tonic pain. In the in vitro assays, these compounds displayed more potent interaction with voltage-gated sodium and calcium channels compared to reference AEDs—phenytoin and carbamazepine. Finally, in the preliminary hepatotoxicity and neurotoxicity studies, compounds **6** and **19** revealed no significant toxicity at a concentration lower than 25 µM. 

## Figures and Tables

**Table 1:** Drug-likeness parameters according to Lipinski and Veber rules.

Compd	Lipinski Rules				Veber Rules	
	MW ≤ 500	Log*p* ≤ 5	NHD ≤ 5 ^a^	NHA ≤ 10 ^b^	NBR ≤ 10 ^c^	TPSA ≤ 140 ^d^
**5**	429.87	2.74	0	4	5	60.93
**6**	429.87	2.73	0	4	5	60.93
**7**	479.88	3.47	0	6	6	60.93
**8**	446.33	2.94	0	3	5	60.93
**9**	446.33	2.95	0	3	5	60.93
**10**	446.33	2.93	0	3	5	60.93
**11**	480.77	3.47	0	3	5	60.93
**12**	480.77	3.48	0	3	5	60.93
**13**	429.87	2.75	0	4	5	60.93
**14**	429.87	2.76	0	4	5	60.93
**15**	479.88	3.45	0	6	6	60.93
**16**	446.33	3.06	0	3	5	60.93
**17**	446.33	2.96	0	3	5	60.93
**18**	446.33	2.97	0	3	5	60.93
**19**	480.77	3.59	0	3	5	60.93
**20**	480.77	3.55	0	3	5	60.93

^a^ NHD: number of hydrogen bond donors; ^b^ NHA: number of hydrogen bond acceptors; ^c^ NBR: number of rotatable bonds; ^d^ TPSA: topological polar surface area.

**Table 2:**
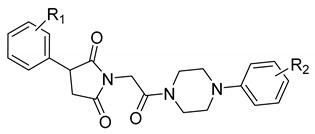
Anticonvulsant activity (MES and 6 Hz tests) and acute neurotoxicity (rotarod test) following i.p. administration of dose 100 mg/kg in mice.

Compd	R_1_	R_2_	Intraperitoneal Administration in Mice		
			MES ^a^	6 Hz ^b^	NT ^c^
**5**	2-Cl	2-F	1/4	1/4	0/4
**6**	2-Cl	4-F	**4/4**	**3/4**	0/4
**7**	2-Cl	3-CF_3_	0/4	**2/4**	1/4
**8**	2-Cl	2-Cl	0/4	0/4	0/4
**9**	2-Cl	3-Cl	0/4	0/4	0/4
**10**	2-Cl	4-Cl	1/4	1/4	0/4
**11**	2-Cl	2,3-diCl	0/4	1/4	1/4
**12**	2-Cl	3,4-diCl	0/4	0/4	0/4
**13**	3-Cl	2-F	1/4	0/4	0/4
**14**	3-Cl	4-F	1/4	1/4	0/4
**15**	3-Cl	3-CF_3_	1/4	**2/4**	0/4
**16**	3-Cl	2-Cl	0/4	nt	0/4
**17**	3-Cl	3-Cl	1/4	nt	0/4
**18**	3-Cl	4-Cl	1/4	nt	0/4
**19**	3-Cl	2,3-diCl	0/4	**2/4**	0/4
**20**	3-Cl	3,4-diCl	0/4	nt	0/4

Ratios where at least two animals were protected or with motor impairment have been highlighted in bold for easier data interpretation. Data indicate number of mice protected or with motor impairment/number of mice tested. The animals were examined 0.5 h after compound administration. ^a^ MES—maximal electroshock seizures test; ^b^ 6 Hz (32 mA)—psychomotor seizure test; ^c^ NT—neurotoxicity–rotarod test; nt—not tested.

**Table 3:** Quantitative pharmacological parameters ED_50_, TD_50_, and PI values in mice (i.p.).

Compd	TPE (h) ^a^	ED50 MES (mg/kg) ^b^	ED50 6 Hz (mg/kg) ^c^	TD50 (mg/kg) ^d^	PI (TD_50_/ED_50_) ^e^
**6**	0.5	68.30 (60.31–77.35)	28.20 (16.86–47.16)	>300	>4.39 (MES) >10.64 (6 Hz)
**19**	0.5	–	124.93 (91.13–191.27)	>300	>2.4 (6 Hz)
**ETX** ^f^	0.25	–	171.74 (141.43–208.52)	440.81 (409.01–475.07)	2.57 (6 Hz)
**VPA** ^f^	0.5	252.74 (220.10–290.22)	130.64 (117.61–145.19)	430.77 (407.92–454.90)	1.70 (MES) 3.30 (6 Hz)

Values in parentheses are 95% confidence intervals determined by probit analysis according to Litchfield and Wilcoxon (1949). ^a^ TPE—time to peak effect; ^b^ ED_50_ (MES—maximal electroshock seizure test); ^c^ ED_50_ (6 Hz, 32 mA, psychomotor seizure test); ^d^ TD_50_ (NT—acute neurological toxicity determined in the rotarod test); ^e^ PI—protective index (TD_50_/ED_50_); ^f^ Reference anticonvulsant drugs ethosuximide (ETX), valproic acid (VPA) tested in the same conditions [15]. A dash indicates not tested.

**Table 4:** In vitro binding assays (concentration 100 μM).

Compound	Na^+^ Channel (Site 2) *	L-type Ca^2+^ (Dihydropyridine Site, Antagonist Radioligand) *
	% Inhibition of Control Specific Binding	
**6**	80.0	82.9
**19**	73.1	35.5
	
PHE	53.9	57.8
CBZ ^a^	17.4	2.6

* Source: rat cerebral cortex. Reference inhibitors: [^3^H]batrachotoxin for the voltage-sensitive sodium channel or [^3^H]nitrendipine for L-type Ca^2+^ channel. Results showing an inhibition higher than 50% are considered to represent significant effects of the test compounds; results showing an inhibition between 25% and 50% are indicative of moderate effect; results showing an inhibition lower than 25% are not considered significant; PHE—phenytoin, CBZ—carbamazepine, ^a^ Data from the previous studies [6].

## Data Availability

The data presented in this study are available in this article.

## Supplementary Materials

The following are available online, Figure S1: Influence of the test compound 6 at a dose of 100 mg/kg on latency time to first clonus in the scPTZ test. Each value represents the mean ± SEM obtained from 4 mice. Statistical analysis: *t*-test: *p* < 0.05., Figure S2: Influence of the test compound 6 at a dose of 130 mg/kg on latency time to first clonus in the scPTZ test. Each value represents the mean ± SEM obtained from 6 mice. Statistical analysis: t-test: NS (not significant).

## Sample Availability

Samples of the compounds **5**–**20** are available from the authors.
