# Supplementary material for: Synthesis, Anticonvulsant, and Antinociceptive Activity of New 3-(2-Chlorophenyl)- and 3-(3-Chlorophenyl)-2,5-dioxo-pyrrolidin-1-yl-acetamides
Source: Molecules. 2021 Mar 12;26(6):1564. doi: 10.3390/molecules26061564 (PMC8000848; doi:10.3390/molecules26061564)

Supplamenatary materials

Synthesis, Anticonvulsant and Antinociceptive Activity of New 3-(2-Chlorophenyl)- and 3-(3-Chlorophenyl)-2,5-dioxo-pyrrolidin-1-yl-acetamides

Małgorzata Góra[1], Anna Czopek[1]*, Anna Rapacz[2]*, Anna Gębska[2], Katarzyna Wójcik-Pszczoła[3], Elżbieta Pękala[3], Krzysztof Kamiński[1]

[1] Department of Medicinal Chemistry, Faculty of Pharmacy, Jagiellonian University Medical College, Medyczna 9 St., 30-688 Krakow, Poland; malgorzata.gora@doctoral.uj.edu.pl (M.G.); anna.czopek@uj.edu.pl (A.C.); k.kaminski@uj.edu.pl (K.K.)

[2] Department of Pharmacodynamics, Faculty of Pharmacy, Jagiellonian University Medical College, Medyczna 9 St., 30-688 Krakow, Poland; a.rapacz@uj.edu.pl (A.R.); anna2.gebska@student.uj.edu.pl (A.G.)

[3] Department of Pharmaceutical Biochemistry, Faculty of Pharmacy, Jagiellonian University Medical College, 9 Medyczna 9 St., 30-688 Krakow, Poland; katarzynaanna.wojcik@uj.edu.pl (K.W.-P.); elzbieta.pekala@uj.edu.pl (E.P.)

Pharmacology

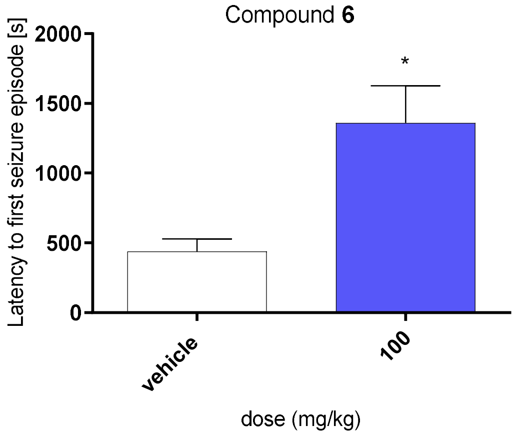

Figure S1. Influence of the test compound **6** at a dose of 100 mg/kg on latency time to first clonus in the *sc*PTZ test. Each value represents the mean ± SEM obtained from 4 mice. Statistical analysis: *t*-test: $p < 0.05$.

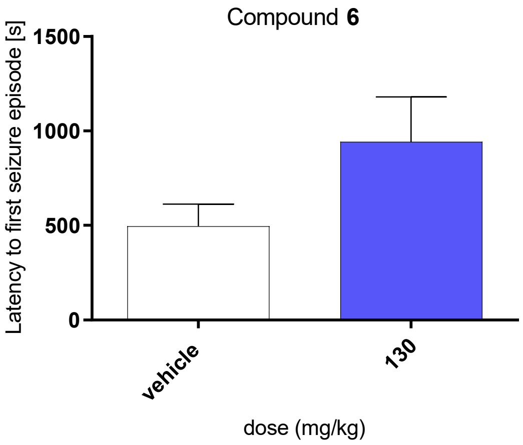

Figure S2. Influence of the test compound **6** at a dose of 130 mg/kg on latency time to first clonus in the *sc*PTZ test. Each value represents the mean ± SEM obtained from 6 mice. Statistical analysis: *t*-test: NS (not significant).

Chemistry

Examples of proton and carbon nuclear magnetic resonance (^{1}H and ^{13}C NMR) spectra for the most active compounds **6**, and **19**, which were recorded on a Varian Mercury spectrometer (Varian Inc., Palo Alto, CA, USA), in $CDCl_3$ operating at 300 MHz (^{1}H NMR) and 75 MHz (^{13}C NMR).

Compound 6

^{1}H NMR

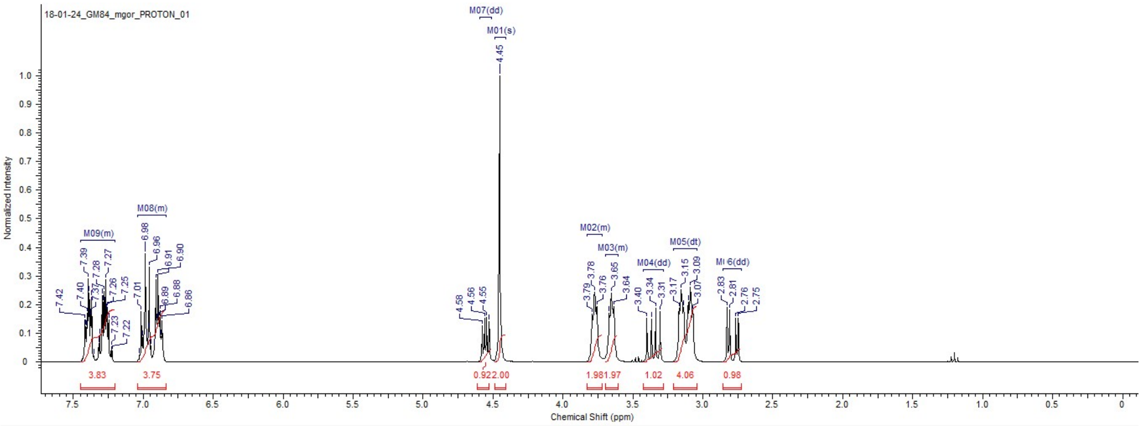

^{13}C NMR

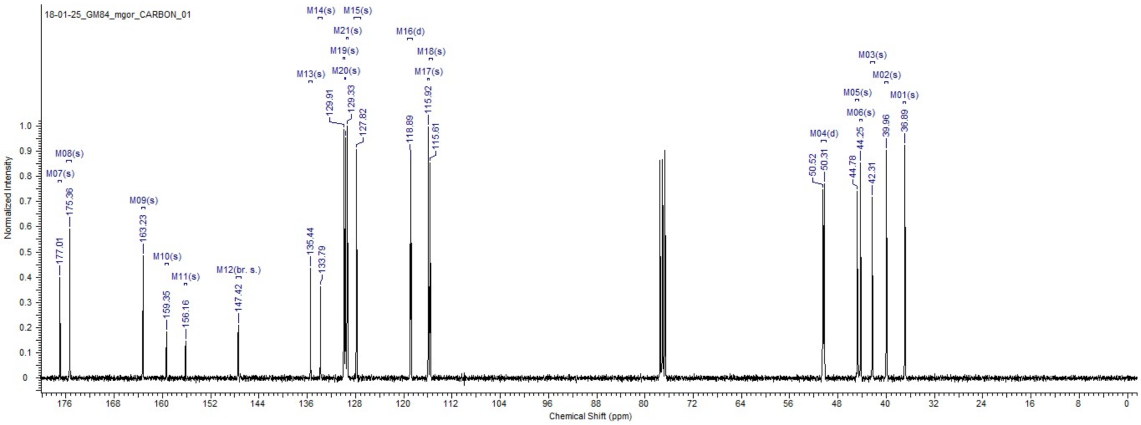

Compound 19

^{1}H NMR

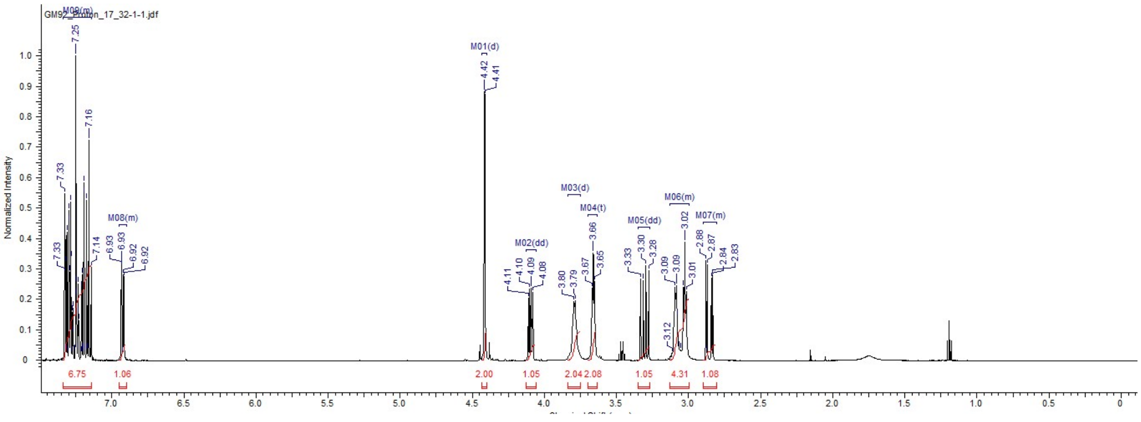

^{13}C NMR

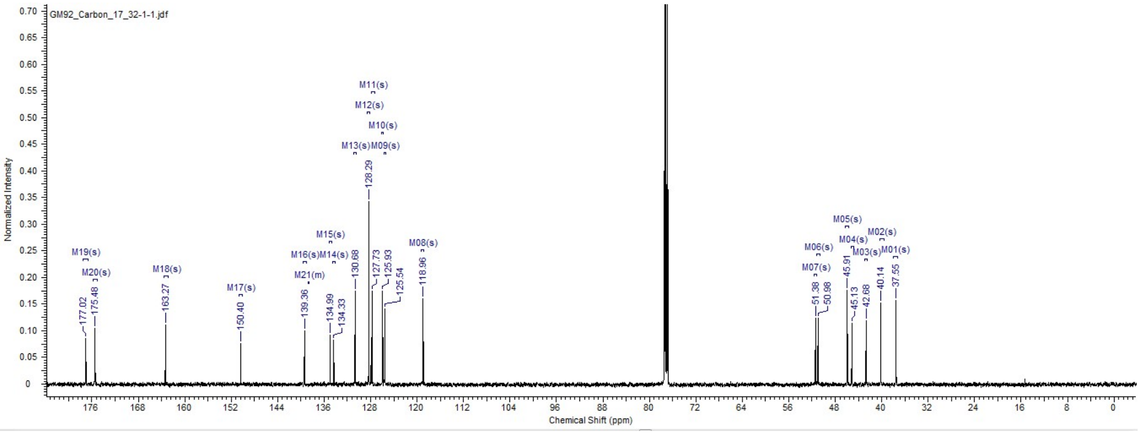

Supplement: Supplementary file 1 [file molecules-26-01564-s001.pdf]
